# The Soul-Sucking Wasp by Popular Acclaim – Museum Visitor Participation in Biodiversity Discovery and Taxonomy

**DOI:** 10.1371/journal.pone.0095068

**Published:** 2014-04-22

**Authors:** Michael Ohl, Volker Lohrmann, Laura Breitkreuz, Lukas Kirschey, Stefanie Krause

**Affiliations:** Museum für Naturkunde, Leibniz-Institut für Evolutions- und Biodiversitätsforschung, Berlin, Germany; Onderstepoort Veterinary Institute, South Africa

## Abstract

Taxonomy, the science of describing and naming of the living world, is recognized as an important and relevant field in modern biological science. While there is wide agreement on the importance of a complete inventory of all organisms on Earth, the public is partly unaware of the amount of known and unknown biodiversity. Out of the enormous number of undescribed (but already recognized) species in natural history museum collections, we selected an attractive example of a wasp, which was presented to museum visitors at a special museum event. We asked 300 visitors to vote on a name for the new species and out of four preselected options, *Ampulex dementor* Ohl n. sp. was selected. The name, derived from the ‘soul sucking’ dementors from the popular Harry Potter books is an allusion to the wasps' behavior to selectively paralyze its cockroach prey. In this example, public voting on a scientific name has been shown to be an appropriate way to link museum visitors emotionally to biodiversity and its discovery.

## Introduction

Taxonomy is an interesting and popular endeavor. It has recently been the subject of a lively discussion on how to improve its standing and perception in science and society [1]–[4]. One of the most important challenges facing taxonomy is how to communicate its importance in solving some of the most important global problems, e.g., health, global food, and conservation [5]. Today, professional taxonomic work, at least in Middle Europe and parts of North America, is mainly conducted in natural history museums, where it is based on enormous comparative collections [6]–[8]. Physical and digital natural history collections are a critical resource for understanding biodiversity and the biodiversity crisis [9]–[11]. This approach is even more important because natural history museums are not only scientific institutions, but also one of the most popular, competent, and successful organizations for the transfer of scientific content to the public. Natural history museums all over the world engage in setting up specific exhibition and presentation formats to communicate current aspects of biodiversity research, conservation, and discovery to the public.

Since the beginning of formal taxonomic description in the 18^th^ century, taxonomic names have been formed on the basis of Latin or Greek words [12]. As a result, at least basic knowledge of classical languages has been perceived as a significant component of taxonomic work [13]. The use of classical languages and rigorous traditional naming procedures contribute to the alienation of the people from this aspect of nature [14]. In past decades, public engagement particularly within natural history museums through citizen science, amateur naturalists, and public exhibitions, has considerably changed the perception, outlook, and application of taxonomy.

It seems to be widely unknown within society that the majority of global species richness still awaits discovery [15]–[17]. At the current pace, about 18,000 new species of organisms are described each year [5]. In the Museum für Naturkunde, Berlin (MfN), one of the largest natural history museums in the world, efforts have been made to engage visitors in the museum's activities in biodiversity research. This includes programs to give visitors insights into the significance and processes of current taxonomic or biodiversity initiatives at the MfN and elsewhere (e.g. the MfN hosts the National Focal Point of the Global Taxonomy Initiative: http://www.gti-kontaktstelle.de/en and was the national organiser of the World Wide Views on Biodiversity in Germany in September 2012: http://www.wwviews-biodiversity.naturkundemuseum-berlin.de/). Here we report on another activity not only to inform visitors about taxonomy, but also to connect people emotionally to biodiversity and its discovery. During a Berlin-wide “Long Night of the Museums” (LNoM) in 2012, we set up several activities in order to inform visitors about the rules, principles and also the pleasures of naming newly discovered species. We also invited museum visitors to vote on the name of an undescribed species of wasp, which we herein formally describe.

## Materials and Methods

The digital images of the holotype were taken with a Canon 6D SLR camera attached to a Leica MZ 12.5 dissecting microscope. Multifocus photographs were focused and captured manually and subsequently stacked using the software package Helicon Focus 5.3.

### Nomenclatural acts

The electronic edition of this article conforms to the requirements of the amended International Code of Zoological Nomenclature, and hence the new names contained herein are available under that Code from the electronic edition of this article. This published work and the nomenclatural acts it contains have been registered in ZooBank, the online registration system for the ICZN. The ZooBank LSIDs (Life Science Identifiers) can be resolved and the associated information viewed through any standard web browser by appending the LSID to the prefix “http://zoobank.org/”. The LSID for this publication is: urn:lsid:zoobank.org:pub:B0F2FFEC-A89E-4A99-B39A-C30C26260B65. The electronic edition of this work was published in a journal with an ISSN, and has been archived and is available from the following digital repositories: PubMed Central, LOCKSS.

Except for *Ampulex dementor* Ohl n. sp., the candidate species names for the public voting listed in Tab. 1 and Tab. 2 are disclaimed for nomenclatural purposes and are not made available by this publication.

**Table 1:** The complete explanatory text concerning the meaning and derivation of each of names.

**A newly discovered digger wasp!**
Help to decide, which scientific name it should bear!
Our newly discovered digger wasp comes from Thailand and belongs in the genus *Ampulex*, which comprises about 130 mainly tropical species. Many species have metallic colors, but our new discovery is a beautifully red-black wasp. As is known from other species of *Ampulex*, it probably moves in a typical running and jumping behavior. Species of *Ampulex* are cockroach-hunters, which make her prey will-less by stinging it right into one of its neural nodes. This allows the wasp to drag her prey in running mode into its nest, just like a zombie.
Michael Ohl and the wasp researchers of the Museum für Naturkunde in his group are eager to describe and officially name this beautiful wasp. Please help to find a suitable name for it!
These are the candidates:
***Ampulex bicolor***: derived from the Latin bi = two and color = color; an allusion to the distinctive, black-red coloration of the wasp.
***Ampulex mon***: The Mon people are one of the earliest known ethnic groups in Thailand. The name is an allusion to the geographic origin of the wasp from Thailand.
***Ampulex dementor***: The species name refers to the dementors, which are fictional characters appearing in Harry-Potter-books. Dementors are magical beings, which can consume a person's soul, leaving their victims as an empty but functional body without personality and emotions. The name is an allusion to the docility of the paralyzed cockroach.
***Ampulex plagiator***: The new species is an ant-mimic: It tries to imitate ants in its general appearance as well as in its way of moving. One can say that the wasp is a plagiarist of the ant, and who is not reminded of current plagiarisms …?

It was handed out to the visitors along with the ballots (originally in German, here translated). In the explanation of the name *Ampulex plagiator*, ‘current plagiarisms’ refers to an at that time widely press-recognized case against a popular politician to have plagiarized his Ph.D. thesis. It could be expected that the majority of visitors from Germany knew this case.

**Table 2:** Proposed species epithets, description of the kind of name and the total and relative number of votes.

proposed species epithet	kind of name	votes	
		total	%
*dementor*	patronym formed after a fictional character from a popular book and movie	105	38.6%
*plagiator*	after a typical feature of the species, but with a humorous connotation	90	33.1%
*bicolor*	after a color character	41	15.1%
*mon*	after a local ethnic group from the geographic origin of the species	36	13.2%
**Total votes**		**272**	

The total number of ballots distributed to visitors was 300, and 272 (90.7%) were returned.

### Terminology

#### Ampulicidae

One of three families of “Sphecidae” (commonly referred to as digger wasps), which are one of the most diverse groups of Aculeata (stinging wasps).

#### Diagnosis

The Diagnosis section of a formal species description includes the characters of the new described species, which distinguishes it from similar and usually closely related species.

#### Etymology

The Etymology section of a species description explains the formation, derivation and grammatical status of the new name.

#### LSID

An acronym for Life Science Identifier. An LSID is a uniform resource name in a specific format to locate pieces of information in the web. In zoological nomenclature, LSID's are used as globally unique identifiers for registration entries in Zoobank, the official registry for zoological nomenclature.

#### Code of Zoological Nomenclature

This is a set of rules and recommendations, which as conventions rule the formal scientific naming, name formation and name handling for organisms treated as animals.

#### Species description

This phrase is used here in terms of the International Code of Zoological Nomenclature as a piece of scientific work, which meets a set of specific criteria to make a new name officially available.

#### Type material

One or more particular specimens, on which a formal species description is based. A holotype is an individual specimen, either the only available specimen or selected out of a series, to which a given species name is attached. If one species is later considered to be composed of actually more than one species, the species, to which the holotype belongs, keeps the previous name.

## Results

### The new species

**Figure 1:**
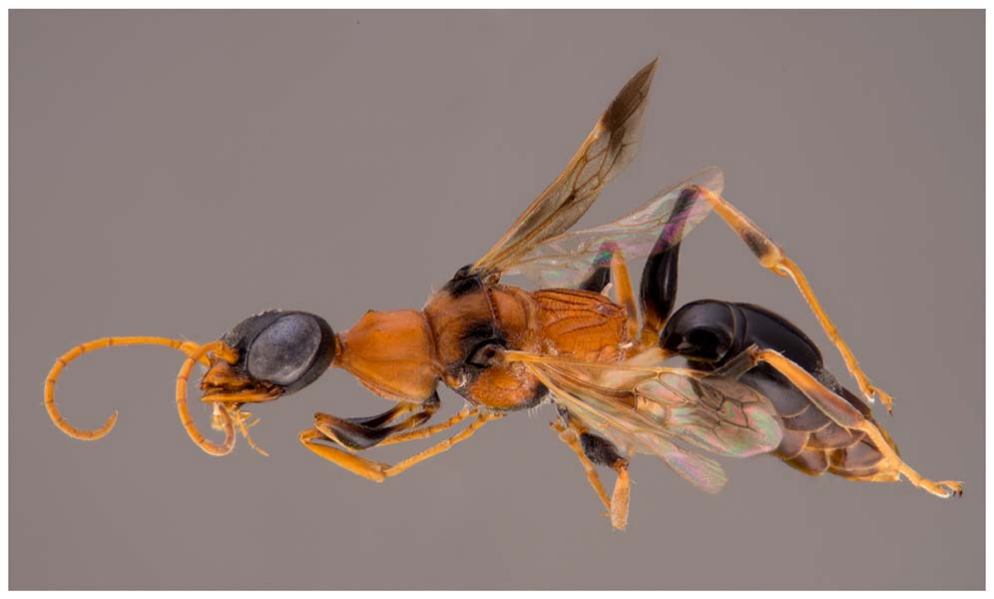
*Ampulex dementor* n. sp., female, holotype, in oblique lateral view. Pin digitally removed from image. Photo: B. Schurian, MfN.

**Figure 2:**
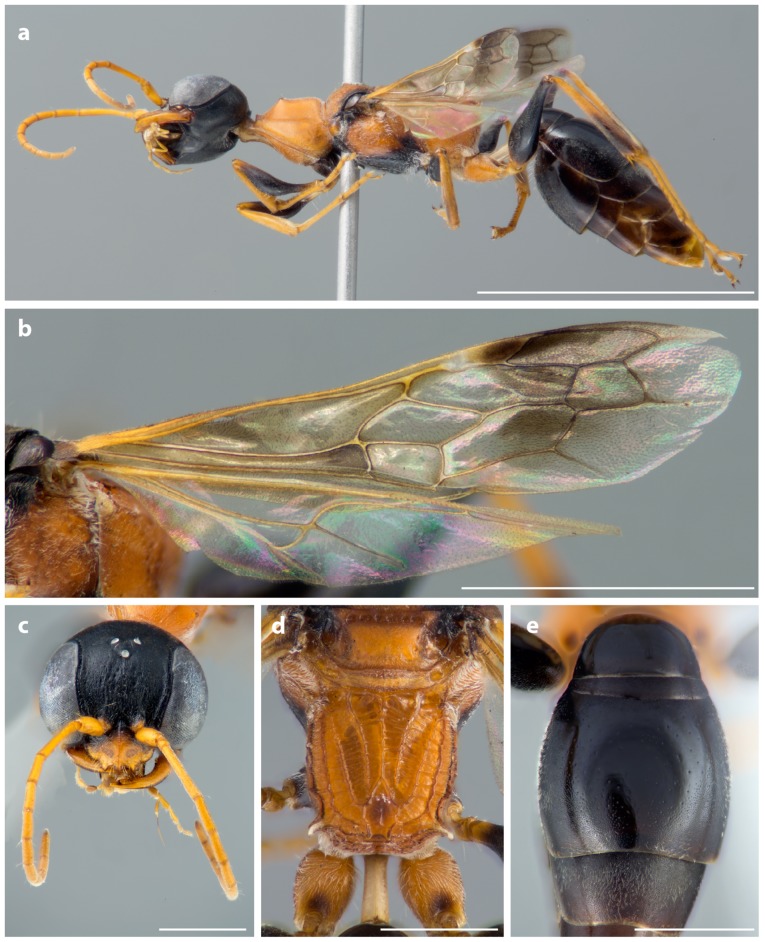
*Ampulex dementor* n. sp., female, holotype. (A) Lateral view. (B) Left fore and hindwings. (C) Head in frontal view. (D) Propodeum in dorsal view. (E) Metasomal terga I-III in dorsal view. Scale bars: (A) 5.0 mm, (B) 2.0 mm, (C-E) 1.0 mm. Photos: B. Schurian, MfN.

Apoid or digger wasps, a diverse group of stinging wasps with about 10,000 species already known [18], are particularly diverse and unexplored in Southeast Asia. Among the many undescribed species of which we are already aware and which are deposited in Berlin's museum collection, we selected a member of the cockroach-hunting wasp genus *Ampulex* Jurine from Thailand in the family Ampulicidae. It belongs to an ant-mimicking group of species with attractive coloration and rather bizarre habitus (Figs 1, 2) and probably also behavior. We expected this genus to attract attention and interest in museum visitors. The specimen was obtained in the course of the TIGER-project (Thailand Inventory Group of Entomological Research), an inventory of Thailand's insects initiated by Michael S. Sharkey from the University of Kentucky, USA. Details of the TIGER-project can be found here: http://sharkeylab.org/tiger/. We have published preliminary results of our analysis of the Thai apoid wasp fauna based on the TIGER material earlier [19].

Ampulicidae, or cockroach wasps, comprise 200 already named species in six genera, which occur mainly in tropical habitats of all continents [20]. With more than 130 species, the genus *Ampulex* is the largest genus in the Ampulicidae [21]. One of its representatives is *Ampulex compressa* (Fabricius, 1781), which has become popular for successfully being cultured in zoos and privately [22]. Its popularity is due to its relatively large body size, beautiful metallic coloration and easily observable and attractive prey stinging and nesting behavior [23].

The new species exhibits some characters unique within the group of species in *Ampulex* with a general ant-like appearance and red-black coloration with marked wings. It is most similar to another species in the genus, described from Sri Lanka (*A. ceylonica* Krombein, 1979). The two have the first two upper plates (terga) of the metasoma (the body section behind the waps-waist) markedly shining, but the sides of the second tergum is dull due to numerous tiny dimples (micropunctures). The two species can be readily distinguished by the vertex, the area of the upper head mainly behind the three simple eyes (ocelli), which is microsculptured and dull in both species, but with only a few indistinct larger punctures in the new species and many, deep and clearly defined punctures in *A. ceylonica*. The two are also different in the morphology of the frons, the head area below the three ocelli. In the new species, the frons has markedly developed longitudinal wrinkles (rugulae) for almost all of its length, whereas in *A. ceylonica*, the frons is indistinctly wrinkled near the lower margin of the frons. Finally, the new species is known from Thailand only, whereas *A. ceylonica* has been exclusively recorded from Sri Lanka.

### The way to the new name

In January 2012, Berlin museums organized the 30^th^ “Long Night of the Museums” (“Lange Nacht der Museen”) (LNoM) (http://archiv.lange-nacht-der-museen.de/30/). Seventy museums all over the city provided guided tours behind the scenes and a wide variety of more than 350 special events. In total, more than 30,000 visitors attended the LNoM, and the MfN alone had 5,226 visitors.

Special events in the MfN comprised guided tours through the scientific collections and various displays in the exhibition hall, which provided information on entomology, geology, history of science, specific information for children and others. We set up an information desk that offered opportunities for visitors to learn about nomenclature. As an example, we demonstrated the basic principles of patronyms by transforming the visitors' personal name into a formal scientific name for a hypothetical species.

On a large video screen we displayed a high-resolution image of a colorful, undescribed species of cockroach wasp of the genus *Ampulex* from Thailand. We informed the visitors, that we intended to describe and name it and that they were asked to vote for the species name to be published. We handed out a ballot with a few explanations on the necessity to select an appropriate name for the newly discovered wasp. In order to avoid large numbers of linguistically incorrectly formed and, thus, inappropriate names, we decided to provide a list of four preselected names. These four names were selected to represent different linguistic kinds of names, but in all cases, we made sure that the names were meaningfully and comprehensibly derived from the species to be described (Tab. 1).

Along with the ballot, we handed out an explanation on the meaning of each of the names. The complete text is given in Tab. 1. The results of the poll are given in Tab. 2.

In addition to the printed program for the LNoM, the MfN informed potential visitors about the possibility to vote for a name of a new animal species in advance on its homepage and using social media like Facebook (https://www.facebook.com/MfN.Berlin) and Twitter (https://twitter.com/MfNBerlin). During and after the LNoM, people were informed that the result would be announced over the same channels.

## Discussion

Society should be concerned not only with the loss of biodiversity but also with its still unexplored richness. Current trends towards an integrative taxonomy, which incorporates a large variety of mainly molecular methods [24], have professionalized and modernized taxonomy. Independent of the technologies employed to delimit species, scientific names, which are based on a common system of nomenclature, are the universal key to biodiversity [25]. ‘When we want to remember something, we give it a name’ [26]. Names are linguistic tags, which are attached to bundles of information, data and hypotheses in order to ease communication about the named entities. Discovering and formally describing species is, thus, a twofold process [27]: the taxonomic research process results in a set of evidence for the hypothetical existence of a certain biological unit, usually a species, which in a subsequent step is formally named. A scientific name and its formal publication have to meet several requirements, which in zoology are organized by the ‘International Code of Zoological Nomenclature’ (http://www.nhm.ac.uk/hosted-sites/iczn/code/). Names as linguistic tags for information on a species are arbitrarily formed, but may have an etymological meaning.

The two parts of the process of species description are fundamentally different and require specific background knowledge to be accessed by non-experts. Species delimitation in modern integrative taxonomy is mostly based on large comparative collections housed in natural history museums, genetic data and morphological information often gained by high-technology imaging systems like μ-computer tomography [28]. The percentage of non-professional taxonomists particularly in entomology is still relatively high [29], but species delimitation and hypothesis formation in taxonomy is a scientifically challenging endeavor and requires scientific knowledge.

In contrast, the naming aspect of taxonomy is different and particularly suitable for communicating the process of species discovery to the public. Based on our experiences, ‘public naming’ of newly discovered species is a suitable means of visitor participation in one of the core disciplines of a natural history museum, the discovery of biodiversity. Today's rules for the formation of scientific names are still partly based on the principles of classical languages, e.g., names are usually Latinized in terms of their endings. It can be expected that the widely decreasing knowledge of classical Latin and Greek in society might serve as a psychological hindrance with regards to engagement in taxonomy. However, it can easily be demonstrated that the formation of names based on modern linguistic elements is an easy task and requires knowledge of very few rules. As an example, we have latinized visitor names together with the visitors to form patronyms.

There are other approaches for involving the public in biological naming processes, which have raised broad publicity. One was a competition for common names of endangered species in Britain, another is the auctioning or selling of naming rights. In 2010, the British newspaper “The Guardian” in collaboration with “Natural England”, a non-departmental public body of the UK government responsible for protecting and developing England's natural environment, and the Oxford University Museum of Natural History, has launched a similar, but in many respects different initiative [30]. The general public was asked to submit self-constructed common names, potentially including humor, national history and any other aspect, for an endangered British click-beetle, *Megapenthes lugens* (Redtenbacher, 1842). The underlying expectation was that such an initiative would raise awareness of a part of Britain's fauna, which is poorly known and often overlooked by the public. The competition was later expanded to the naming of ten endangered species of organisms in Britain, and the judging panel received more than 3,000 entries. The results were made public by “The Guardian” only a few weeks later, and the naming initiative has been received as a way to build up “a cultural connection that will help their conservation cause” [31]. The major difference to our approach is that the public could be asked to construct totally new names out of colloquial language, because it was a competition for common names. Scientific name forming is different in being at least partly based on classical languages, which cannot expect the public to be familiar with, and in the need being compliant with the rules of nomenclature. Therefore, we decided to propose a list of candidate names to prevent uncontrolled submission of formally unsuitable names. This also differs from our approach in that the British common name initiative had a panel of judges, rather than the general public, select the final name. In our approach, we chose a variety of names and the public voted for the one they preferred. However, the case of the “Queen's executioner beetle”, which was the winning name, is another mechanism for connecting biodiversity to the general public.

Since the 17^th^ century, new species have frequently been named after sponsors of the expedition which found the unnamed specimens or after other people supporting the authors' research. In this context, dedicated species names have always been exploited to bring the sponsors' name to the public in order to bind sponsors to the organisms named after them and, often more relevant, to the describing researcher or to the conservation project in the relevant area. Naming species after sponsors has always been accompanied by the expectation that the sponsor will continue funding the project or the scientist. From here it is only a short step to sell or auction naming rights [32]. As an example, the discoverer of a new species of tit monkey from the Madidi National Park in Bolivia auctioned off the naming right in a blind internet auction on the internet, which received significant publicity. The auction was won by the Internet Casino Goldenpalace.com with a bid of US$ 650.000. They chose the common name Golden Palace Monkey, which was latinized and formally published as *Callicebus aureipalatii* Wallace, 2005. The entire sum of money raised was provided to the Bolivian Park Service to run the Madidi National Park. Due to the large amount of money and a casino involved, this unusual example of auctioning naming rights has received much attention from the public. Many taxonomists around the world have successfully auctioned naming rights for their newly discovered species, most of them for significantly smaller amounts of money. In 1998, a German non-profit initiative named “BIOPAT” set up an internet broking platform for selling naming rights [33]. Taxonomists can present their new species to the public and offer potential donors the opportunity to sponsor a newly discovered animal or plant species and of giving this a scientific name of their own choice. Half of the money raised is provided to support nature conservation initiatives in the country of the origin of the new species, and half is used to promote the describer's further research activities in taxonomy. The ethical implications of “selling” names are still a controversial issue among taxonomists [32]. Besides the ethical implications, there is also a barrier for most people to participate because of the cost. Thus, selling a name will unlikely link as many people emotionally to a single new species as the method employed here.

Our public voting of a taxonomic name was received very positively. Visitors were highly interested and during the event spent a significant amount of time asking for details and listening to explanations. We prepared 300 ballots in advance, of which about 90% were returned (Tab. 2). Even this is already a remarkable high return rate, many more visitors were interested in participating. Upon request, visitors clearly indicated a personal bond to the species to be named and curiosity regarding this unexpected opportunity to participate in the naming process. Even more, some visitors also pointed out that having been involved in the naming process of the new wasp species had changed their personal perception of insects and this unexplored part of nature in a positive way. It has been shown previously, that a positive general perception of previously ignored or even rejected organisms can add to calls for better conservation management [34]. We are aware that visitors to a natural history museum are a biased population, because they are more likely to be interested in natural history, have higher education levels, and to be aware of the relevant content [35]. However by creating direct opportunities for participation in the discovery of new species for the public, we help visitors feel like partners and co-owners of the content of the museum [36] and the global ‘Catalog of Life’. Although we have here selected a species with attractive outlook and behaviors, we are convinced that a similar public approach would be applicable to any organism as long as it is presented in an attractive manner. In summary, our public name voting shows that public engagement in natural history museums, through citizen science, amateur naturalists, public activities and participation, can contribute to bringing the perception, outlook, application, and appreciation of taxonomy back to its roots – the people's science.

## Taxonomy

### Description of *Ampulex dementor* Ohl n. sp

Figures 1 and 2.

#### Etymology

The new species is named after the “dementors”, which are fictional characters of the wizarding world in the popular “Harry Potter” book series by the writer Joanne K. Rowling. These creatures are said to suck out every good feeling, every happy memory and the free will of anybody getting too near.

The dementor's fictional behavior and effects reminded us of the effect of the stinging behavior of *Ampulex* on the behavior of its cockroach prey. After being stung by the wasp, specific behaviors of the cockroach are inhibited (e.g. escape behavior) while others are unaffected (e.g. locomotion). The wasp grabs the partly paralyzed cockroach by one of the antennae and guides it to a suitable oviposition location, the prey following the wasp in a docile manner. This is a unique strategy of behavioral modulation of a prey by a wasp's sting [23], [37].

#### Diagnosis

*Ampulex dementor* belongs in a group of species, which are characterized by black head and metasoma in combination with a mostly red mesosoma (Figs 1–2). They are slender, relatively small, and they are assumed to be ant-mimics. As in most other species in this group, *A. dementor* has partly clouded forewings (Fig. 2A, B) and markedly long legs, which are adapted for the typical running-jumping behavior of many *Ampulex*. The new species also has long, curved whitish posterolateral propodeal spines (Fig. 2D), which are present in most other species of the group except for *A. ruficornis* (Cameron, 1889). *Ampulex dementor* is most similar to *A. ceylonica* Krombein, 1979 from Sri Lanka: Both species have shining terga I and II (Fig. 2E), with the sides of tergum II densely micropunctate and dull. They can be distinguished by the evenly microsculptured and vertex in *A. dementor*, with only a few, shallow and indistinct macropunctures. The vertex is also evenly microsculptured in *A. ceylonica*, but with many, dense, sharply defined macropunctures. Additionally, the frons of *A. dementor* bears irregular, longitudinal rugulae, with a shallow, shining carina along the midline, which completely reaches from the clypeus to the midocellus (Fig. 2C). In *A. ceylonica*, the frons is indistinctly wrinkled toward the clypeus, but lacks distinct rugulae, except for a short, shining carina along the midline for the ventral one-third of the frons. *Ampulex dementor* is known only from Thailand.

#### Description

Female (male unknown). Total length (excluding appendages) 9.6–10.9 mm (n = 2).

Black, the following light red: mandible, most of clypeus, prothorax, mesothorax (except for the subalar area), the posterolateral corners of mesoscutum and the ventral surface of the mesopleuron beneath omaulus and sternaulus, upper half of metapleuron, propodeum, most or all of hindcoxa and all trochanters, tibiae (except distal half of hindtibia), all tarsi; propodeal spines and petiole whitish, metasomal segments IV and V dark brown, VI pale brown. Forewing slightly yellowish, with dark marking in marginal cell, distal half of first and complete second submarginal cells, and distal half of discoidal cell II, and small infumate spot at apex of submedial cell. Hindwing hyaline.

Head elongate behind eyes, completely microsculptured, dull. Frons with irregular, longitudinal rugulae and with shallow, shining carina along midline, entire from clypeus to midocellus. Vertex with a few, irregularly scattered, shallow punctures. Gena impunctate. Mandible unmodified. Compound eye surrounded by carina, which is strongest at level of frons, diverging above, lower interocular distance across toruli about 0.75x upper interocular distance across hindocelli. Flagellomere I slightly shorter than II and III combined.

Pronotum microsculptured, impunctate, with sharp, median furrow and a pair of acute tubercles anterolaterally. Mesonotum micropunctate, shining, with irregularly interspersed macropunctures, notauli irregularly punctate, mesopleuron similar, macropunctures subcontiguous on upper half, 1–2 puncture diameter apart on lower half, sternaulus und omaulus coarsely punctate, scutelleum similar to mesonotum, with a few macropunctures posterolaterally. Propodeal dorsum coarsely areolate, with a median and four lateral longitudinal ridges, connected by several tranverse carinae, with truncate median process at posterior margin, posterolateral spines long, slender, curved, propodeal sides above metapleural sulcus and posterior propodeal declivity coarsely areolate, propodeal side below metapleural sulcus shining.

Forewing submarginal cell bent away from wing margin for most of its length, pointed distally, continued by a non-pigmented vein to wing tip. First intersubmarginal veinlet lacking, so that submarginal cells I and II fused to form a markedly long cell. First recurrent vein meeting combined submarginal cells I and II almost medially, second recurrent vein meeting (apparent) submarginal cell II in basal one-fourth. Hindwing media diverging at cu-a.

Legs long and slender, femora markedly swollen medially (fore and midlegs) or basally (hindleg). Tarsomeres IV with ventral mat of fine pubescence, short, tarsomeres V inserted dorsally at base of IV.

Petiole almost evenly tubular, about as long as tergum I in dorsal view. Tergum I with sides diverging toward tergum II, not nodose, with constriction between terga I and II. Tergum I and II shining, virtually impuncate, except for densely, finely punctate posterolateral corner of II. Punctation of tergum III finer and denser than on posterolateral corner of tergum II, becoming increasingly indistinct on following terga. Sternum II markedly convex, with strong transverse groove subbasally, very finely, densely micropunctate. Following sterna microsculptured. Metasomal segments V and VI compressed laterally.

#### Material examined

Holotype, female. T2448. Thailand, Phetchabun, Khao Kho NP, mix deciduous forest, 16°32.539′N 101°2.483′E, 524 m, pan traps, 10–11 June 2007, S. Chachumnan & S. Singtong (Queen Sirikit Botanic Garden, Chiang Mai, Thailand).

Paratype: same data as holotype, except for: T2447, 9–10 June 2007 (1 female, Museum für Naturkunde, Berlin, Germany).

### Additional material studied

#### Ampulex ceylonica Krombein

Kan. Dist., Udawattakele Sanct., 2 and 9 Feb 1979, det. K.V. Krombein (1 male, 1 female, paratypes, Smithsonian Institution, Washington, USA)

*Ampulex ruficornis* (Cameron): Thailand, Phuket, det. M. Ohl (1 male, 1 female, Museum für Naturkunde, Berlin); Sri Lanka, Colombo, det. K.V. Krombein (1 male, 1 female, Smithsonian Institution, Washington, USA)

#### Ampulex sp

37 specimens of *Ampulex* from Thailand and Singapore belonging to the ant-mimicking group with black-red coloration have been studied, some of which have been provided by the TIGER-project and are currently deposited in the Museum für Naturkunde, Berlin, Germany, and some are in the collection of the Smithsonian Institution, Washington, USA. They probably belong to three or four undescribed species, but some of them are singletons. Whereas the identity of *Ampulex dementor* as an undescribed species can be safely deduced from the original description and the examination of available material of the other known species in the group, the status of the unidentified material is less clear and can only be elucidated after studying the types of some of the species from Southeast Asia described by Cameron and Tsuneki.

### Persistent identifiers

Life Science Identifiers (LSID), issued by Zoobank (http://www.zoobank.org):

Author: urn:lsid:zoobank.org:author:878259F2-C3C6-4264-B04A-C397E01E5C8E

Publication: urn:lsid:zoobank.org:pub:B0F2FFEC-A89E-4A99-B39A-C30C26260B65

Species-group name: urn:lsid:zoobank.org:act:1358BB4F-FCA2-4463-A11C-E063A55C7B15

Uniform Resource Identifiers (URI), issued by the Museum für Naturkunde, Berlin (MfN), linking to type and drawer images of the digital collection of the MfN:

Holotype: http://coll.mfn-berlin.de/u/MfN_Hym_Amp_I00001

Paratype: http://coll.mfn-berlin.de/u/MfN_Hym_Amp_I00002

MfN insect drawer containing the paratype: http://coll.mfn-berlin.de/u/MfN_Hym_Amp_D0003

## References

[1] Wheeler QD (ed.) (2008) The New Taxonomy. The Systematics Association Special Volume Series 76. Boca Raton: CRC Press. 256 p.

[2] Wheeler QD, Knapp S, Stevenson DW, Stevenson J, Blum SD, et al (2012) Mapping the Biosphere: Exploring Species to Understand the Origin, Organization and Sustainability of Biodiversity. Syst Biodiv 10: 1–20.

[3] Padial JM, Miralles A, De la Riva I, Vences M (2010) The integrative future of taxonomy. Front Zool 7: 16 doi:10.1186/1742-9994-7-16 20500846 10.1186/1742-9994-7-16 PMC2890416

[4] Sluys R (2013) The unappreciated, fundamentally analytical nature of taxonomy and the implications for the inventory of biodiversity. Biodiv Conserv 22: 1095–1105.

[5] Costello MJ, May RM, Stork NE (2013) Can we name earth's species before they go extinct? Science 339: 413–416. 23349283 10.1126/science.1230318

[6] Lohrmann V, Vohland K, Ohl M, Häuser C (eds) (2012) Taxonomische Forschung in Deutschland: Eine Übersichtsstudie. Berlin: Museum für Naturkunde. Available: http://www.biodiversity.de/images/stories/Downloads/taxo-studie-01-2012.pdf. Accessed: 2014 April 1.

[7] Boxshall G, Self D (2011) UK Taxonomy & Systematics Review – 2010. Available: http://webarchive.nationalarchives.gov.uk/20110707232615/http://www.nerc.ac.uk/research/programmes/taxonomy/documents/uk-review.pdf. Accessed: 2014 April 1.

[8] Lovejoy TE, Brouillet L, Ford Doolittle W, Gonzalez A, Green DM, et al. (2010) Canadian Taxonomy: exploring biodiversity, creating opportunity. Council of Canadian Academies, Ottawa, Canada. Available: http://www.scienceadvice.ca/uploads/eng/assessments%20and%20publications%20and%20news%20releases/biodiversity/biodiversity_report_final_e.pdf. Accessed: 2014 April 1.

[9] Natural Sciences Collections Association (NatSCA) (2005) A Matter of Life and Death. Natural science collections: why keep them and why fund them? Available: http://natsca.org/sites/default/files/publications-full/A-Matter-Of-Life-And-Death.pdf. Accessed: 2014 April 1.

[10] Shaffer HB, Fisher RN, Davidson C (1998) The role of natural history collections in documenting species declines. TREE 13: 27–30. 21238186 10.1016/s0169-5347(97)01177-4

[11] Causey D, Janzen DH, Townsend PA, Vieglais D, Krishtalka L, et al (2004) Museum Collections and taxonomy. Science 305: 1106–1105. 10.1126/science.305.5687.1106b 15326336

[12] Mayr E (1982) The Growth of Biological Thought. Cambridge, MA: Belknap Press.

[13] Winston JE (1999) Describing Species – Practical taxonomic Procedure for Biologists. New York, USA: Columbia University Press. 512 p.

[14] Yoon CK (2009) Naming Nature – The clash between instinct and science. New York, USA: Norton. 352 p.

[15] Chapman AD (2009) Numbers of Living Species in Australia and the World, 2^nd^ edition. Australian Biological Resources Study, Canberra. Available: http://www.environment.gov.au/system/files/pages/2ee3f4a1-f130-465b-9c7a-79373680a067/files/nlsaw-2nd-complete.pdf. Accessed: 2014 April 1.

[16] Mora C, Tittensor DP, Adl S, Simpson AGB, Worm B (2011) How many species are there on earth and in the ocean? PLoS Biology 9, (e1001127) , doi:10.1371/journal.pbio.1001127 10.1371/journal.pbio.1001127 PMC3160336 21886479

[17] Costello MJ, Wilson S, Houlding B (2012) Predicting total global species richness using rates of species description and estimates of taxonomic effort. Syst Biol 61: 871–883. 21856630 10.1093/sysbio/syr080

[18] Aguiar AP, Deans AR, Engel MS, Forshage M, Huber JT, et al.. (2013) Order Hymenoptera. In: Zhang, ZQ, editor. Animal Biodiversity: An outline of higher-level classification and survey of taxonomic richness (Addenda 2013). Zootaxa 3703. pp. 51–62. 10.11646/zootaxa.3703.1.1 26146682

[19] Lohrmann V, Kirschey L, Krause S, Schulze M, Ohl M (2012) TIGER-wasps – A preliminary review of the apoid wasp diversity in Thailand (Hymenoptera: Apoidea). Mitt DGaaE 18: 31–34.

[20] Ohl M, Spahn P (2010) A cladistic analysis of the cockroach wasps based on morphological data (Hymenoptera: Ampulicidae). Cladistics 26: 49–61. 10.1111/j.1096-0031.2009.00275.x 34875746

[21] Pulawski WJ (2014) Sphecidae. Number of Species. Available: http://research.calacademy.org/sites/research.calacademy.org/files/Departments/ent/sphecidae/Number_of_Species.pdf. Accessed: 2014 April 1.

[22] Veltmann J, Wilhelm W (1991) Husbandry and display of the Jewel wasp, *Ampulex compressa*, and its potential value in destroying cockroaches. Int Zoo Yb 30: 118–126.

[23] Libersat F (2003) Wasp uses venom cocktail to manipulate the behavior of its cockroach prey. J Comp Physiol [A] 189: 497–508. 10.1007/s00359-003-0432-0 12898169

[24] Dayrat B (2005) Towards integrative taxonomy. Biol. J. Linn. Soc. 85: 407–415.

[25] Thompson FC (1996) Names: The keys to Biodiversity. In: Reaka-Kudla, DML, Wilson, E, Wilson, EO, editors. Biodiversity II. Washington: National Academies Press. pp. 199–216.

[26] Bowker GC (2005) Memory practices in the sciences. Cambridge, MA: MIT Press. 280 p.

[27] Ohl M (2007) Principles of Taxonomy and Classification - Current procedures for naming and classifying organisms. In: Henke, W, Tattersall, I, editors. Handbook of Palaeoanthropology. Vol. 1 . Heidelberg: Springer-Verlag. pp. 141–166.

[28] Faulwetter S, Vasileiadou A, Kouratoras M, Dailianis T, Arvanitidis C (2013) Micro-computed tomography: Introducing new dimensions to taxonomy. Zookeys 263: 1–45. 10.3897/zookeys.263.4261 PMC3591762 23653515

[29] Fontaine B, van Achterberg K, Alonso-Zarazaga MA, Araujo R, Asche M, et al.. (2012) New species in the Old World: Europe as a frontier in biodiversity exploration, a test bed for 21st century taxonomy. PLoS ONE 7, (e36881) , doi:10.1371/journal.pone.0036881 10.1371/journal.pone.0036881 PMC3359328 22649502

[30] Environmental editor (2010) The clicking, larvae-eating beetle: *Megapenthes lugens* The Guardian, 25 June 2010. Available: http://www.theguardian.com/environment/2010/jun/25/name-a-species-megapenthes-lugens. Accessed: 2014 January 10.

[31] Monbiot G (2010) Ten British species now have an identity we care about. The Guardian, 16 July 2010. Available: http://www.theguardian.com/environment/georgemonbiot/2010/jul/16/name-a-species-winners. Accessed: 2014 April 1.

[32] Behrer P (2009) Value of a name: Naming rights auctions and their potential for conservation finance. Student Essay for the “2009 Conservation Leadership Dialogue on Conservation Capital in the Americas). Available: http://www.conservationcapitalintheamericas.org/Patrick%20Behrer%20-%20Value%20of%20a%20Name.doc. Accessed: 2014 April 1.

[33] BIOPAT (2013) BIOPAT: Patrons for Biodiversity. Available: http://www.biopat.de/englisch/index_e.htm. Accessed: 2014 April 1.

[34] Simpfendorfer CA, Heupel MR, White WT, Dulvy NK (2011) The importance of research and public opinion to conservation management of sharks and rays: a synthesys. Marine Freshw Res 62: 518–527.

[35] Evans EM, Spiegel AN, Gram W, Frazier BN, Tare M, et al (2009) A conceptual guide to natural history museum visitors' understanding of evolution. J Res Sci Teach 47: 326–353.

[36] Simon N (2010) The Participatory Museum. Santa Cruz: Museum 2.0. Available: http://www.participatorymuseum.org/read/. Accessed: 1 April 2014.

[37] Haspel G, Rosenberg LA, Libersat F (2003) Direct injection of venom by a predatory wasp into cockroach brain. J Neurobiol 56: 287–292. 12884267 10.1002/neu.10238

